# Neuromodulatory Effects of Adjunctive High Definition Transcranial Direct Current Stimulation (HDtDCS) on Auditory Verbal Hallucinations in Schizophrenia Patients: A Sham Controlled Diffusion Tensor Imaging Study

**DOI:** 10.1192/bjo.2022.213

**Published:** 2022-06-20

**Authors:** Apurba Narayan Mahato, Sanjay Kumar Munda, Alok Pratap

**Affiliations:** Central Institute of Psychiatry, Ranchi, India

## Abstract

**Aims:**

To see the neuromodulatory effects of adjunctive HD-tDCS on white matter connectivity by using Diffusion Tensor Imaging (DTI) in schizophrenia patients with Auditory Verbal Hallucinations (AVH)

**Methods:**

This was a randomized, double blind, sham controlled study. 40 patients of schizophrenia with prominent auditory verbal hallucinations and 10 age sex matched healthy controls were selected. The patients were randomly assigned to 2 groups and were given active or sham adjunctive HDtDCS (Active Treatment = 10 sessions of 2 mA current applied for 20 minutes, twice daily for 5 days, at left temporo-parietal Junction (TPJ); Sham treatment = 10 sessions of 1 mA current, twice daily for 5 days was applied for 30 sec at left TPJ). Fractional anisotropy of left arcuate fasciculus by Diffusion tensor imaging was assessed and severity of schizophrenia symptoms and auditory hallucinations were rated on PANSS and PSYRATS-AH at baseline, after 1st week (i.e. end of HDtDCS sessions) and 4 weeks after the end of the HDtDCS sessions). Patients received stable dose of antipsychotics for the total study duration (equivalent to or more than 400 mg of chlorpromazine) to eliminate confounding bias. Fractional anisotropy of left arcuate fasciculus by Diffusion tensor imaging was assessed in healthy controls. DTI data were analysed by DSI Studio software. Statistical analysis was done by SPSS version 25.

**Results:**

1. Both the patient groups were comparable with regard to socio-demographic variables and baseline clinical variables.
2. There was no significant difference in the values of Fractional Anisotropy in Left Arcuate Fasciculus among the patients and healthy controls at baseline.
3. The group receiving active adjunctive HDtDCS, showed significant improvement in the frequency domain of AVH over time, in time*group comparison by repeat measure ANOVA with Mauchly's test of sphericity and Greenhouse-Geisser correction [p = 0.011 and partial eta square = 0.129].
4. There was no significant difference in change in the Fractional anisotropy of the left arcuate fasciculus noted between the groups over time.
5. Application of HDtDCS was not associated with significant side effects, minor itching and mild burning sensation being the only reported side effects

**Conclusion:**

Adjunctive active HD-tDCS to the left temporo-parietal junction showed a statistically significant improvement in frequency of auditory verbal hallucinations (AVH) in schizophrenia patients, when compared to sham stimulation.

